# Careful Examinations

**Published:** 1872-05

**Authors:** 


*Careful Examinations.*—In the same journal, Dr. W. E. Driscoll writes as follows:

"While cutting away a cavity of decay, recently, in a manner similar to the system advocated by Dr. Arthur in his new work on the 'Prevention of Decay of the Teeth,' I was more than ever impressed with a sense of our *duty* in making examinations of the teeth; that is, to use the utmost care in our power to detect decay at its very inception, if so presented to us.

I had polished the approximal surface of the adjoining cuspid and examined it with a magnifying glass. Neither myself nor two or three other persons who happened to be watching the operation could see any sign of decay. But noticing that the surfaces of the two teeth would come together too flatly soon after the wedge was withdrawn, I proceeded to bevel off the cuspid so that a narrow edge would rest against the incisor near the labial surface.

"It was then that I made the surprising discovery that, although there was no indication of decay on the *polished* surface, for a space corresponding to a No. 4 bur drill, the dentine was not only very much softened, but also somewhat discolored. Having cut down to the bottom of this decay, so as to be imperceptible from a front way, and polished it, it was left in a condition to be easily cleansed, and consequently safe from decay. There can be no doubt, of course, that this decay was caused by a fissure in the enamel, so small as to escape detection.

"Although this case may be a better illustration of the value of Dr. Arthur's system, than the necessity of greater than usual care in examinations, I am willing that it should do both, and to that end I have called attention to this circumstance."

